# PROPENSIX: pressure garment therapy using compressive dynamic Lycra^®^ sleeve to improve bi-manual performance in unilateral cerebral palsy: a multicenter randomized controlled trial protocol

**DOI:** 10.1186/s13063-022-06041-1

**Published:** 2022-02-05

**Authors:** A. Gerard, M. Toussaint-Thorin, Y. Mohammad, G. Letellier, S. Fritot, S. Masson, A. Duhamel, C. Donskoff, Y. Zagame, L. Beghin, L. Gottrand

**Affiliations:** 1 Service de Soins de Suites et Réadaptation pédiatrique, CHU Reims, AMH 47 Rue Cognacq-Jay, F-51092 Reims, France; 2 Service d’Éducation et de Soins Spécialisés à Domicile APF, 50 Square Frédéric Chopin, F-60175 Creil, France; 3 Maison de rééducation et d’autonomie, 20 Rue Anatole France, F-95260 Beaumont-sur-Oise, France; 4 Établissement de Santé pour Enfants et Adolescents de la région Nantaise, 58 Rue des Bourdonnières, F-44200 Nantes, France; 5 Médecine Physique et de Réadaptation Pédiatrique, CHU Amiens, 1 Rue du Professeur Christian Cabrol, F-80054 Amiens, France; 6 Établissement de Soins de Suite et Réadaptation Pédiatrique Marc Sautelet APF, 10 Rue du Petit Boulevard, F-59650 Villeneuve-d’Ascq, France; 7 CHU Lille, ULR 2694-Metrics: évaluation des technologies de santé et des pratiques médicales, University of Lille, 2 Avenue Oscar Lambret, F-59000 Lille, France; 8 Service de Soins de Suite et de Réadaptation Pédiatrique, Centre Paul Dottin, 26 Avenue Tolosane, F-31522 Ramonville-Saint-Agne, France; 9 Medical Z, 14 Rue Georges Cuvier, F-37550 Saint-Avertin, France; 10 CHU Lille, CIC1403 - Clinical Investigation Center, University of Lille, 2 Avenue Oscar Lambret, F-59000 Lille, France; 11 Institut d’Éducation Motrice pour enfants et adolescents Christian Dabbadie APF, 64 Rue de la Liberté, F-59650 Villeneuve-d’Ascq, France

**Keywords:** Cerebral palsy, Upper limb, Children, Splint, Orthotic device, Randomized controlled trial, Placebo, Pressure garment therapy, Compressive dynamic Lycra^®^ sleeve, Bimanual performance

## Abstract

**Background:**

Upper limb impairment affects activity and participation in children with unilateral cerebral palsy (UCP). Pressure garment therapy (PGT) using compressive dynamic Lycra^®^ garments is an innovative intervention proposed for the management of cerebral palsy consequences. The PROPENSIX study aims to evaluate the efficacy of a therapy using a Lycra^®^ sleeve as compared to a placebo sleeve to improve bi-manual performance measured by the Assisting Hand Assessment (AHA) in children with unilateral cerebral palsy.

**Methods:**

The PROPENSIX trial is a multicenter, prospective, placebo-controlled, double-blinded, randomized study. One hundred children with UCP, aged from 5 to 10, are randomly assigned as soon as they are recruited in a 1:1 ratio to perform usual daily activities, especially activities involving bimanual performances, with Lycra^®^ sleeve or placebo sleeve during 6 months. The primary endpoint is the change in bimanual performance from inclusion to 6 months, evaluated by AHA. The secondary endpoints evaluate changes from inclusion to 6 months in other dimensions of the International Classification of Functioning (ICF), upper limb movement capacity assessed by Quality of Upper Extremity Skill Test (QUEST), and health-related quality of life evaluated by Pediatric Quality of Life Inventory 3.0 Cerebral Palsy Module (PedsQLTM 3.0 CP Module) and in body structures and functions domain assessed by neuro-orthopedic examination and somatosensory evoked potentials (SEP).

**Discussion:**

The PROPENSIX study is the largest randomized controlled trial (RCT) aiming to evaluate the efficacy of a PGT using compressive dynamic Lycra^®^ sleeve in UCP. Enhancement of children’s bimanual performance at the end of the 6 months wear of the Lycra^®^ sleeve should improve evidence regarding this type of treatment and expand discussion about their recommendation in clinical practice. Data from secondary outcomes assessments should bring interesting arguments to discuss the Lycra^®^ sleeve action on mobility, tonus, and sensory impairments in children with unilateral cerebral palsy.

**Trial registration:**

ClinicalTrials.govNCT02086214. Retrospectively registered on March 13, 2014

**Trial status:**

Study start data: December 2012. Recruitment status: completed. Primary completion date: April 2021. Estimated study completion date: December 2022. Protocol version 10 (date: February 2018).

## Introduction

Cerebral palsy is a neurodevelopmental disorder caused by nonprogressive lesions in the immature brain occurring before, during, or after birth. It is the most common physical disability in childhood, with a stable prevalence of 2 per 1000 live births [1]. Unilateral cerebral palsy (UCP) represents 20 to 30% of spastic cerebral palsy [2].

Arm and hand dysfunctions are the main problems in UCP. These troubles depend on several factors, including the extent of sensory loss, severity of paresis, degree of spasticity or presence of retractions, and whether or not dystonic movements are present [3]. It is estimated that 40 to 90% of children with spastic UCP have impaired somatosensory function [4,5]. Correlations between the magnitude of somatosensory dysfunction and movement impairments have been established [6,7]. Abnormal processing of somatosensory stimuli is suspected to contribute to poor cortical feedback during probabilistic learning of movement, providing imprecise or incorrect inferential data [8–10].

Cerebral palsy affects every dimension of the International Classification of Functioning (ICF). This framework is now guiding the evaluation and management of the children’s disability. Upper limb dysfunction is considered to be the main impairment that limits activity and restricts participation in hemiplegic children. Recent reviews and meta-analyses provide evidence-based arguments to guide the management of cerebral palsy [11–13]. The most frequently cited interventions for upper limb management are constraint-induced movement therapy (CIMT), bimanual training, interventions using new technologies (virtual reality and computer-based training therapy intervention), and botulinum toxin combined with specific interventions [14]. Splinting is one of the usual treatment strategies in cerebral palsy. Static, semi-dynamic, and dynamic splints are used among several purposes: prevent deformities, improve posture and movement, and facilitate functional performance [15].

Among dynamic splints, pressure garment therapy (PGT) is an innovative therapy using compressive dynamic Lycra^®^ garments. Lycra^®^ is a synthetic elastane fiber used in the confection of tailor-made close-fitting garments. The Lycra^®^ fabric is used to create a constant pressure and deliver a neutral heat on the concerned body part. Mechanical properties of Lycra^®^ garments have been established in studies involving healthy and hemiplegic adult subjects [16,17]. In children with cerebral palsy, previous studies advocate that pressure garment therapy (PGT) using compressive dynamic Lycra^®^ garments can improve postural alignment, joint stability, and movement efficiency and can enhance posture, balance, coordination, gross motor function, hand function, and gait of children with cerebral palsy and other health conditions [18–23]*.* The interest for using compressive dynamic Lycra^®^ garments reside in the fact that they are soft in nature and provide support while allowing movement.

Several mechanisms are proposed to explain how Lycra^®^ garments act. They are thought to decrease spasticity by prolonged stretch and cutaneous stimulation from tight skin contact which provides neutral heat. By decreasing hypertonia in spastic muscles, Lycra^®^ sleeve utilization on the impaired upper extremity should allow a better control of antagonist muscles (elbow and wrist extensors for example). In addition, the homogenous pression applicated by Lycra^®^ garments can modulate the sensory input and improve the sense of joint position and body awareness by stimulating mechanical receptors [20,24]. Despite nearly 30 years of research, pressure garment therapy has a low level of evidence in cerebral palsy management. One main reason is the lack of large randomized controlled trials (RCT). More studies are needed, especially high-quality studies focusing on functioning, in all dimensions of the ICF perspective.

In children with asymmetric impairments, wearing a compressive dynamic Lycra^®^ sleeve could bring a better utilization of the impaired arm, facilitate bi-manual coordination, and support learning of better motor patterns. Wearing this device while practicing bimanual activities (during school, playing, and eating activities) should result in a better improvement in bi-manual performance than practicing bi-manual activities alone. Inherent properties of Lycra^®^, such as homogenous compression, applied on the impaired upper limb of children should allow them to perform more accurate motor patterns, notably by an action on movement sensory-motor integration.

PROPENSIX study aims to evaluate efficacy of a PGT using a compressive dynamic Lycra^®^ sleeve as compared to a placebo sleeve to improve bi-manual performance measured by the Assisting Hand Assessment (AHA) in children with UCP.

## Methods and design

Table 1 summarizes the trial registration data and information about the study design and population.

**Table 1:** Trial registration data, design of the study, and enrollment criteria

Data category	Information
Primary registry	ClinicalTrials.gov: NCT02086214, registered on March 13, 2014
Secondary identifying numbers	ANSM ID-RCB number: 2011-A01129-32 Ethics committee: CPP 12/05
Sponsor	Lille University Hospital
Contact	Maison Régionale de la Recherche Clinique, CHU Lille, Boulevard du Professeur Leclercq, F-59037 LILLE CEDEX FRANCE Tel. : 03 20 44 68 91 Mail : ciclille@chru-lille.fr
Short title	PROPENSIX study
Scientific title	PROPENSIX: Pressure Garment Therapy using compressive dynamic Lycra^®^ sleeve to improve bi-manual performance in unilateral cerebral palsy, a multicenter randomized controlled trial
Country of recruitment	France
Inclusion criteria	Unilateral cerebral palsy (perinatal or antenatal etiology), 5 to 10 years old, social insurance, written informed consent
Exclusion criteria	Allergy to Lycra^®^, contra-indication to pressure therapy (e.g., skin lesions, allergic contact dermatitis), behavior or speech troubles, Botulinum Neurotoxin received within the 4 preceding months on the impaired arm, tutorship or curatorship, predictable lack of compliance
Intervention	Treatment: Lycra^®^ sleeve (Medical Z^®^, pressure = 15 to 25 mmHg) Placebo: Placebo sleeve (Medical Z^®^, pressure < 5 mmHg)
Study type	Interventional
Study design	Prospective, randomized, placebo-controlled, parallel assignment, double-blinded, multicenter, superiority trial
Target sample size	100
Primary outcome	Performance, evaluated by AHA (Time frame: 6 months)
Secondary outcomes	Capacity, evaluated by QUEST (Time frame: 6 months) Body structures and functions, evaluated by SEP and neuro-orthopedic examination (Time frame: 6 months) Participation, evaluated by PedsQL^TM^ 3.0 CP Module (Time frame: 6 months)
Estimated primary completion date	April 2021

*AHA* Assisting Hand Assessment, *QUEST* Quality of Upper Extremity Skill Test, *PedsQL™ 3.0 CP Module*: Pediatric Quality of Life Inventory 3.0 Cerebral Palsy Module

### Ethical considerations and trial registration

This clinical trial is approved by the Ethics Committee of Lille (Comité de Protection des Personnes Nord-Ouest IV, Lille University Hospital, N° CPP 12/05) and the French competent authorities (ANSM, N° 2011-A01129-32). It is registered at ClinicalTrials.gov under identifier NCT02086214 [25].

According to the Declaration of Helsinki, written informed consent is obtained from both parents of the child before enrollment. On the consent form, participants will be asked if they agree to use their data should they choose to withdraw from the trial. Participants will also be asked for permission for the research team to share relevant data with people from the Universities taking part in the research or from regulatory authorities, where relevant. This trial does not involve collecting biological specimens for storage. The consent form and information material are written in French and are available on request, from the corresponding author.

This protocol follows French regulations regarding biomedical research on medical devices. The trial sponsor did not judge necessary to establish a Data Safety Monitoring Board regarding the absence of specific risk. Serious adverse reactions are collected during the whole course of the trial and systematically reported to the principal investigator and safety department of the clinical trial sponsor. Any modification to this protocol is agreed by the ethics committee before implementation and notified to the health authorities in accordance with local rules.

### Trial design

PROPENSIX is a multicenter, prospective, double-blinded, randomized, placebo-controlled superiority trial using a medical device. The seven French study centers involved are specialized in pediatric rehabilitation. Both parents/children and physicians/therapists are blinded regarding the type of sleeve (active or placebo) used during the whole duration of the trial. Un-blinding will be performed for statistical analysis since it is needed for safety outcome comparison between the groups. Primary aim statistical analysis will be also un-blinded as adherence level is mandatory for future intention-to-treat and per-protocol statistical analysis.

Enrollment into the PROPENSIX study started in December 2012 and has been completed since September 2020. The clinical phase of the study was completed in April 2021. The coding process (neurologic exams, AHA, QUEST, and quality of life tests) and data handling are ongoing until the end of 2022.

Subjects are enrolled by the investigator of each study center. The randomization is centralized. The randomization sequence was provided by an independent statistician (who did not take part in assessing the patients at any point in the study) using computer-generated random numbers structured in blocks. The block size information is not specified in the protocol to ensure that investigators are not able to anticipate treatment arm assignment. The randomization ratio is 1:1. The randomization list is maintained by the sponsor. It has been communicated to the splint manufacturer, Medical Z^®^, ensuring a randomized allocation of the sleeves to each included child, on condition of anonymity.

### Sample size estimates

Based on the literature [26,27], the mean value of AHA was estimated at 1.8 logit (standard deviation = 2) in the placebo splint group. In the Gordon study [27], an improvement of 48% was obtained with a hand-arm bimanual intensive therapy administrated during 1 month. Based on this study and considering the duration of our therapy (6 months), we expected an improvement of 60% in favor of the Lycra® sleeve (mean AHA = 0.72 logit). Considering a two-sided Student test without adjustment, 53 patients per arm were needed (power 80%, alpha = 5%). The analysis will be adjusted for baseline AHA value, allowing to improve the design efficiency. Assuming a 0.4 correlation between the 2 measures (baseline and post-treatment) corresponding to a relative efficiency of 1.16, 45 patients were required. One hundred patients (50 in each arm) were planned to consider a 10% rate of non-analyzable data.

### Population

#### Patient’s eligibility

The inclusion criteria are children with ante-natal or peri-natal hemiplegic cerebral palsy, aged 5 to 10, having social insurance, written informed consent. The exclusion criteria are allergy to Lycra^®^, contra-indication to pressure therapy (e.g., skin lesions, allergic contact dermatitis), behavior or speech troubles, treatment with Botulinum Neurotoxin on the involved arm within the preceding 4 months, children under tutorship or curatorship, and predictable lack of compliance.

Patients may be discontinued from this trial at any time, firstly for voluntary discontinuation. Other specific reasons for discontinuing a patient are the administration of Botulinum Neurotoxin on the involved arm during the study and/or compliance under 80%. Any discontinuation is referred as soon as possible to the principal investigator. Data regarding the discontinued patients will be analyzed in the intention-to-treat analysis.

#### Demographic data

The following demographic data are collected for each participant: age, sex, impaired side, medical history, and current treatments (usual medications, botulinum toxin injections, rehabilitation therapies). The presence of unilateral neglect, presence, and type of cognitive troubles and level of intelligence quotient (IQ) are also collected, as well as the level of scholarship and adaptations needed (special needs assistant for example).

Functional profile of the child is classified using Gross Motor Function Classification Scale (GMFCS) [28] and Manual Abilities Classification System (MACS) [29]. GMFCS is commonly used to describe the gross motor function, notably because it has strong discriminative validity [30]. It focuses on describing gross motor function in self-initiated movements and, in particular, during sitting and walking. The performances of the child are classified according to five levels of functions, from level I which designates independent movement to level V which designates complete assistance. MACS has been developed to categorize how children with cerebral palsy can use their hand when handling objects in daily activities. It particularly points out the child’s use of both hands together and typical manual performance, in opposition to his/her best manual capacity. Similar to GMFCS, MACS consists of five levels which are intended to be clinically meaningful.

### Outcomes

#### Primary outcome: performance measure, Assisting Hand Assessment

The variation of the Assisting Hand Assessment (AHA) from baseline to 6 months is the primary outcome. The AHA is a standardized and criterion-referenced test [31]. It evaluates the performance of children when using their impaired upper extremity during bimanual activities. It reflects what the child really does in his/her daily activities. It is widely used in cerebral palsy evaluation, both in clinical and research purposes. The affected hand is designated as the assisting hand.

The AHA must be administered and scored by a certified occupational therapist. It consists of a 10–15 min video-recorded and semi-structured play session. The child is seated and presented a selection of standardized toys. The scoring is made on the video record, focusing on how the affected hand is used together with the non-affected hand. The detailed criteria are given in the test manual. Quality of performance is scored on a 4-point scale (4 = effective, 3 = somewhat effective, 2 = ineffective, 1 = does not do) for 22 items. Items are divided into 6 categories: general use, arm use, grasp and release, fine motor adjustments, coordination, and pace. In addition, the specific criteria describing behaviors within the categories are defined for each item. Items describe different types of object-related actions of the assisting hand. The total AHA raw score, ranging from 22 (low ability) to 88 (high ability), can be converted into a logit score and a logit-based 0–100 scale based on Rasch analysis [32]. According to Krumlinde [32], the results are expressed in “logit-based AHA-unit” [32]. AHA is a validated assessment, with good inter- and intra-rater reliability (respectively 0.97 and 0.99) [33] and a good sensitivity to change [34].

#### Secondary outcomes

For all secondary outcomes, we will consider the variation between the inclusion (baseline value) and post-treatment value (6 months).

##### Capacity measure: Quality of Upper Extremity Skill Test

Quality of Upper Extremity Skill Test (QUEST) is used to assess upper limb quality of movement [35]. This validated instrument is a criterion-referenced measure. It contains 33 items divided into 4 domains: dissociated movement, grasp, weight-bearing, and protective extension. Some items are detailed into sub-items. Both upper extremities are assessed following a dichotomous scale (2 = able to complete item, 1 = not able to complete item). Administration and scoring last 30 to 45 min. Items scores are summed, and formulas are used to obtain percentages for each domain. Domains percentages are summed and divided by number of domains to obtain a total score which is expressed in percentage. A greater score indicates better capacity of the upper limb.

The QUEST is widely used, both in clinical practice and as a standardized outcome measure in studies evaluating treatments’ efficacy. It is an interesting instrument because it measures a combination of impairments and function. It is used to assess the quality of upper limb movement, but it also measures components of hand function and provides information about movement and postural responses. Parametric studies report an adequate to high inter- and intra-rater reliability in 18 months to 12 years old children [36–40].

##### Participation measure: Pediatric Quality of Life Inventory 3.0 Cerebral Palsy module

To describe children’s quality of life, parents are asked to answer the Pediatric Quality of Life Inventory 3.0 Cerebral Palsy module (PedsQL™ 3.0 CP Module, Parents Report) [41]. It is a brief, simple and valid questionnaire, intended to measure health-related quality of life in a population of children and adolescents with cerebral palsy. It assesses the child’s quality of life among 7 domains: daily activities, school activities, movement and balance, pain and hurt, fatigue, eating activities, and speech and communication. The completion time is 5 min. It is administrated following PedsQL™ Administration Guidelines. The French version used in this trial has been translated by MAPI™ Research Institute.

##### Body structures and functions measure: neuro-orthopedic examination

Neuro-orthopedic examination is detailed and scored in a standardized way. The examination focuses on mobilities of the impaired arm: flexion and abduction of the shoulder, flexion and extension of the elbow, pronation and supination of the forearm, flexion and extension of the wrist, flexion and extension of the fingers, and abduction of the thumb. Examiner assesses the passive range of motion at high and low speed and gives a score of Modified Ashworth Scale (MAS) for each mobility cited. Active range of motion is recorded for the same mobilities.

The results obtained after this examination are scored so that changes can be analyzed. At the first examination, each mobility is scored on a binary scale (0 = normal range of motion, 2 = presence of anomaly). The results of the second examination, 6 months later, are scored in comparison with the first examination, on a 0 to 3 scale (0 = normal range of motion, 1 = lower anomaly, 2 = stable anomaly, 3 higher anomaly).

On the sensory level, examination details the type of trouble (epicritic, thermoalgesic, and/or proprioceptive defect) during a comparative examination. Both sides impairments are recorded. Examiner reports the absence or presence of a deficit.

##### Body structures and functions measure: somatosensory evoked potentials

Based on our hypothesis that motor improvement in impaired limb may be subtended by changes in somatosensory function, we wanted to assess the somatosensory system with an objective measure. Somatosensory evoked potentials (SEP) seem complementary to the clinical examination of sensory perception for two main reasons. In children with UCP under the age of 10, changes in sensory function are difficult to measure consistently using behavioral responses to stimuli [42]. Evoked related potentials responses could detect treatment-induced changes prior to their consistent appearance in behavioral measures, particularly in young children populations [8].

SEP are elicited by electrical stimulation of the median nerve at the wrist, using a 5 channels Synergy Medelec system (Oxford Instruments Medical®). The median nerve is stimulated percutaneously at the anterior face of the wrist (cathode is proximal, anode is distal). Intensity stimulation is minimal intensity causing painless muscular contraction in thenar muscles (usually around 8 mA). Each stimulation block consists of 150 stimuli (frequency is 1 Hz; single stimulus duration is 0.1 ms). The stimulation block can be repeated up to 5 times to obtain the best valid record.

The child is lying down on an examination table in a semi-darkened quiet room. He is encouraged to relax using music, comforter, or parental presence if needed. The recording is performed if the child is sufficiently relaxed. If not, it is delayed, and other attempts are made later on.

The recording electrodes are placed over the following locations, at various levels of the nervous system. One is placed at Erb’s point (ipsilateral to the stimulus) to record N9 potential. One is placed over the sixth cervical spine process to record N13 potential. One is placed on the scalp at C3 (left hemisphere) or C4 (right hemisphere), following the ten-twenty electrode system, to record P14, N20, P27, P45, and N30 potentials. The reference electrode is A1 or A2 on the contralateral ear. Ground electrode is placed on the stimulated arm, on a proximal position as compared to stimulation electrodes.

The analysis period is 50 ms at Erb’s point and cervical point, 100 ms at scalp points. SEP are amplified with a band pass fiber at 3–1000 Hz. Electrode impedance is kept under 10 kOhm. Latency of N9, N13, P14, N20, P27, P45, and N30 of each side are recorded for the study. Calculated measures are N13–N20 interval representing the conduction from dorsal horn of the spinal cord to cortex and P14–N20 interval representing the conduction from sub-cortical level to cortex (brain conduction time). Ratios of delay conduction are calculated as follows: delay of the affected side divided by delay of the unaffected side. Amplitudes of N20–P27 are also calculated to allow the calculation of the amplitude ratio (amplitude of affected side divided by amplitude of unaffected side).

##### Safety outcome

Safety outcome will be evaluated during the 6 months wearing period of PGT Lycra^®^ sleeve upper arm. Safety outcomes will include the number and intensity of adverse events of interest (AEIs). AEIs will be defined as adverse event imputable to compressive therapy and Lycra^®^ wearing, localized at the arm. AEIs will be classified into two subgroups: (i) cutaneous events linked to Lycra^®^ wearing and (ii) compression events linked to compressive therapy. According to the device classification panel from FDA regulation, the PROPENSIX PGT Lycra^®^ sleeve is a class I medical device. In this context and according to French regulations, it will not be mandatory to address a Medical Device Reporting process and a Data Safety Monitoring Board report to conduct this study. To perform this safety assessment, parents will be asked to daily report AEIs and other problems occurring during the Lycra^®^ sleeve pressure garment during wearing period on a parental self-report diary logbook. The diary logbook will be carefully checked by the investigator before the physical examination at each hospital visit (i.e., 3 months and 6 months) using a structured interview. AEIs will be classified at posteriori by the investigator using Bégaud et al. classification (minor/moderate/serious) usually used for drug clinical trial report [43]. Cutaneous events were defined as itchy contact dermatitis, red skin rash, and spots; compression events will be defined as mechanical swelling, arm pain, “Blue hand,” tingling, discomfort, sore thumb, and tightness complaint from a child. The frequency of AEIs will be computed as a percentage of occurred days of AEIs from the total wearing time.

Systolic blood pressure (SBP) and diastolic blood pressure (DBP) will also be measured as a safety outcome using an oscillometer device with a pediatric bladder.

##### Level of adherence to Lycra^®^ sleeve pressure garment procedure

The level of adherence will be assessed using a conventional paper-based method (Lillo-Navarro C). The diary logbook will collect the daily amount of Lycra® sleeve pressure garment wearing period in number of hours and the reason of non-adherence if the sleeve wearing is under 3 h per day. The level of adherence will be expressed in the percentage of number of days when sleeve wearing reaches at least 3 h per day compared to the length of duration in days (start and end date of wearing period). The frequency of reasons of non-adherence will be computed as a percentage of occurred days of AEIs from the total wearing time.

### Procedures

#### Intervention

##### Lycra^®^ sleeves

**Fig. 1:**
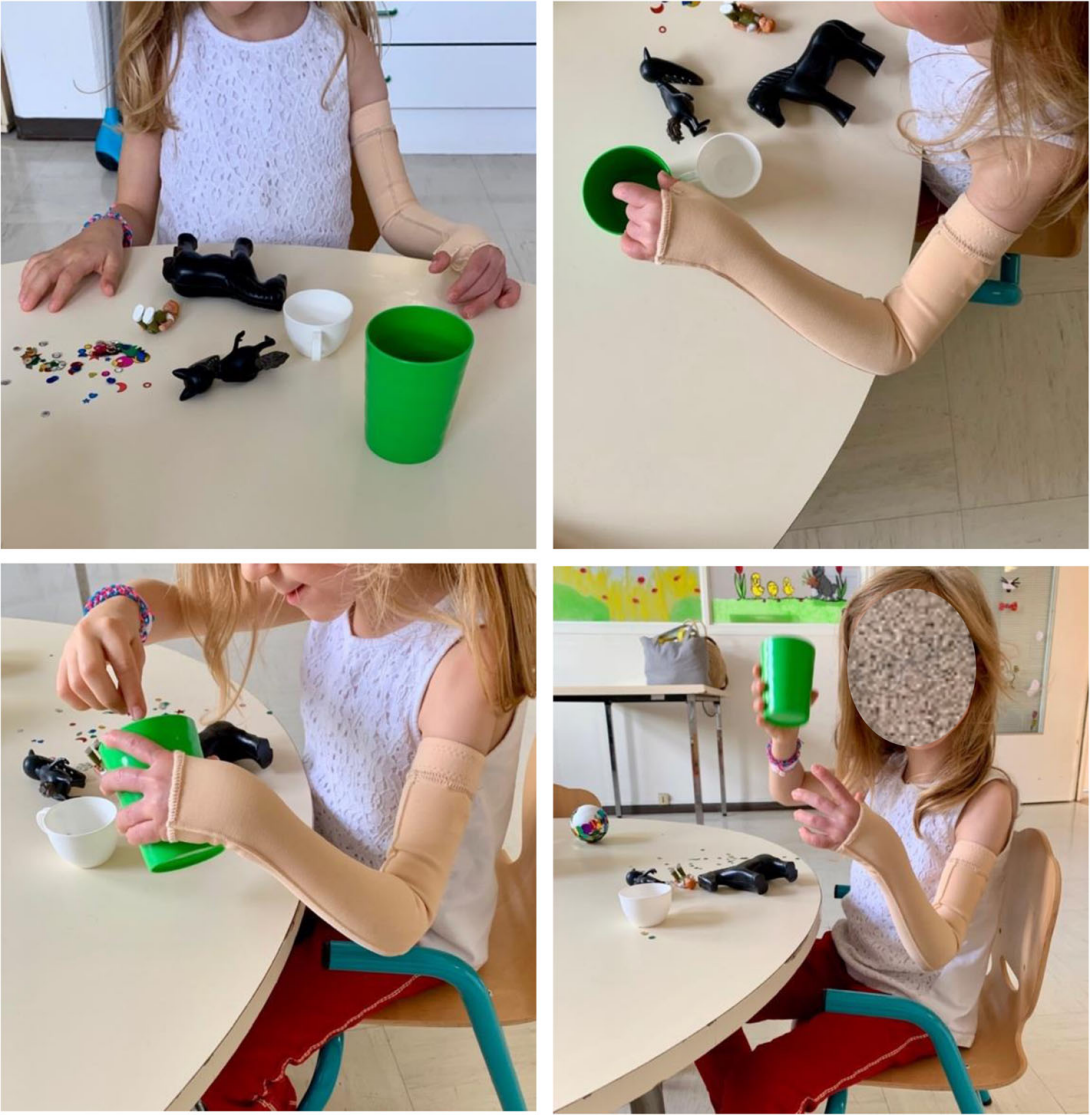
A child wearing a Lycra^®^ sleeve while playing

Lycra^®^ sleeves used in this trial are manufactured by Medical Z^®^ [44]. They are tailor-made sleeves which covers the arm from the axilla to half of the palm and the thumb, without covering other fingers (Fig. 1). The compressive dynamic Lycra^®^ sleeve, also denominated as “active Lycra^®^ sleeve,” and the placebo sleeve have the exact same appearance. No distinction between an active sleeve and a placebo sleeve can be made by investigators or patients. The active sleeve generates a homogeneous pressure ranging from 15 to 25 mmHg. The placebo sleeve provides a pressure under 5 mmHg. The sleeve notice specifies conditions of utilization: the sleeve has to be worn on the impaired arm, on bare skin, avoiding wrinkles by adjusting it from the hand to the axilla. It also provides cleaning and care precautions, as well as safety advices.

##### Daily activities

After enrollment, the child is asked to wear the sleeve (active or placebo) at least 3 h a day, every day, for 6 months. The sleeve has to be worn during usual daily activities, especially in activities involving bimanual performances, and during rehabilitation sessions. Each patient’s therapists (physiotherapists and occupational therapists) are informed of the child enrollment in a trial and asked to follow instructions of harmonization for rehabilitation, but there is no major modification of the usual rehabilitation care.

Written general recommendations are provided to guide rehabilitation:

- Stimulation of proprioceptive function: analytic proprioception stimulation, ground bearing and weight-bearing transfers, installation quality and symmetry, mirror feedback, and dynamic proprioception stimulation (opposition and pushing games, moving of heavy objects).
- Stimulation of active mobility, on proximal and distal levels of the hemiplegic side, with static shoulder and arm, hand aiming, approach, and grip and release exercises.
- Stimulation of bimanual coordination during daily activities, developing assisting hand capacities, and passing from one hand to the other.

Additional personalized recommendations are added, regarding the child’s state of development and actual capacities of the upper extremity.

#### Conduct of the trial

##### Multicenter trial

The trial takes place in 7 French pediatric rehabilitation centers. Patients are recruited in medical or medico-social structures that usually hosts children with cerebral palsy and are situated in the local areas of the involved centers. Technical and organizational support is provided by the Clinical Research Center of Lille University Hospital (Centre d’Investigation Clinique, CIC-1403_CHU-Inserm de Lille).

##### Study visits

**Fig. 2:**
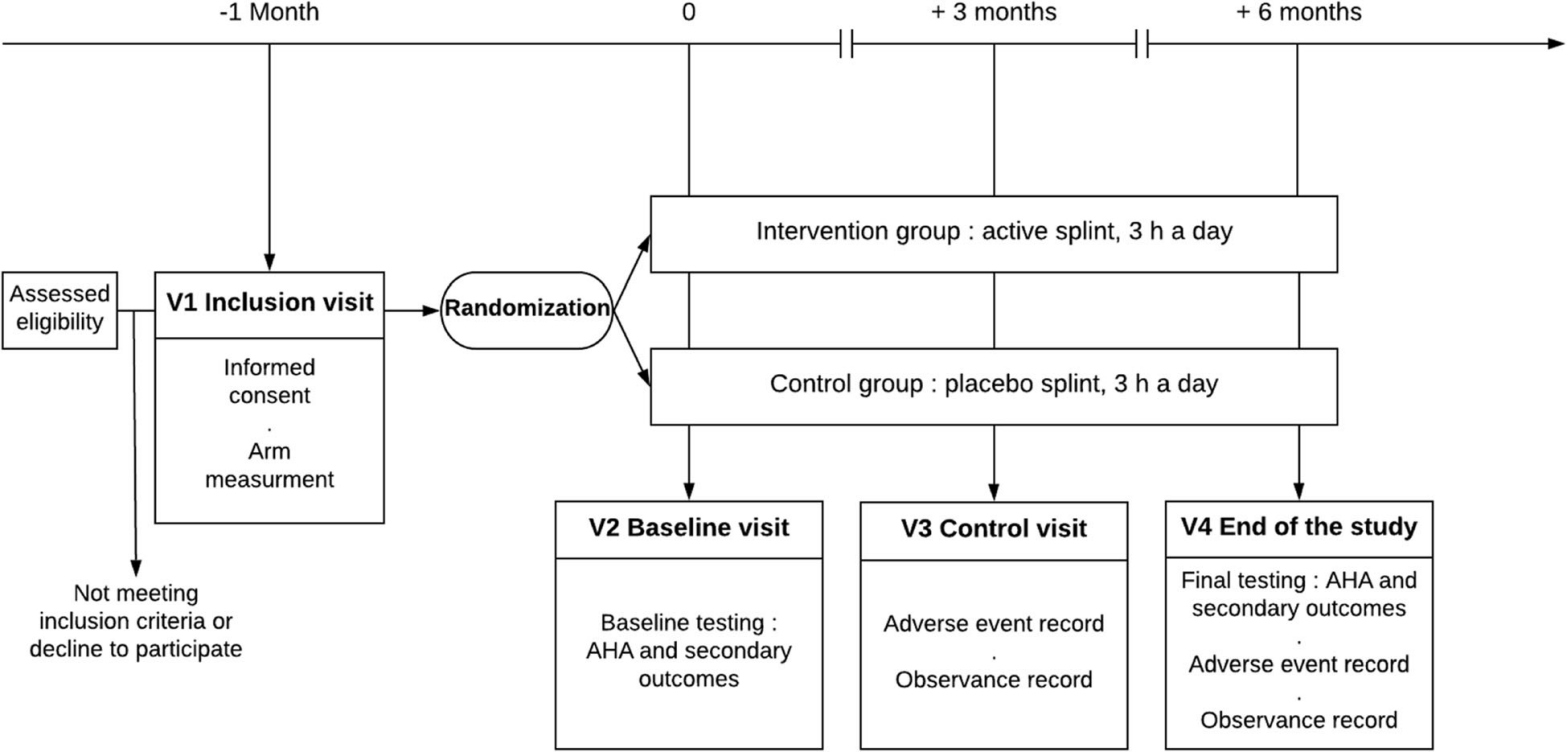
Experimental design. AHA, Assisting Hand Assessment

There are 4 study visits (V1 to V4). Visit duration is 2 h for V1 and V3 and 4 h for V2 and V4. The study schedule is presented in Table 2. Flow of participants and timeline are presented in Fig. 2.

**Table 2:** Schedule for data recording

	Inclusion visit, V1 (− 1 month)	Baseline visit, V2 (0 months*)	Control visit, V3 (3 months*)	End of study, V4 (6 months*)
Information and consent	X			
Standard examination		X	X	X
Neuro-orthopedic examination		X		X
Inclusion and exclusion criteria verification	X			
Randomization	X			
Weight, height, blood pressure, pulse	X**	X	X	X
Arm measurement	X			
AHA***		X		X
QUEST***		X		X
SEP***		X		X
PedsQL™ 3.0 CP Module		X		X
Adverse reactions report			X	X
Compliance evaluation			X	X

*AHA* Assisting Hand Assessment, *QUEST* Quality of Upper Extremity Skill Test, *PedsQL™ 3.0 CP Module* Pediatric Quality of Life Inventory 3.0 Cerebral Palsy Module

*+ 2 weeks

**Weight and height only

***Assessments without wearing the splint

After giving their consent, participants are recruited and randomized during the inclusion visit (V1) which happens 1 month before the beginning of the intervention. The occupational therapist or investigator, specifically formed, takes precise measurements of the child’s arm, following the manufacturer’s instructions. The investigator sends the measurement data to Medical Z®, so that the confection of the sleeve can begin by the manufacturer according to randomization list (active sleeve or placebo sleeve), on condition of anonymity. The sleeve will be delivered by the manufacturer to the study center within 15 + 3 days. The investigator delivers the tailor-made sleeve (active or placebo) to the child and parents during the baseline visit (V2), with oral and written instructions regarding its utilization and care. In addition, the investigator edits the recommendations for rehabilitation harmonization, destinated to therapists involved in the child’s usual care. The sleeve is reclaimed at the end of the study. Involved therapists, investigators, patients, and their parents are blinded regarding the type of sleeve (active or placebo) received by the child according to randomization.

Outcomes evaluation occurs 1 month after inclusion, during the baseline visit (V2), and at the end of the intervention, after 6 months of daily wear of the Lycra^®^ sleeve, and during the final visit (V4). Baseline and final testing are realized without wearing the splint. AHA, the primary outcome, is assessed by a certified occupational therapist. QUEST is assessed by the same experienced occupational therapist, following the QUEST manual [35]. Neuro-orthopedic examination is conducted by the investigator following the report form of the study. SEP are recorded by an experienced neurophysiology technician. PedsQL™ 3.0 CP Module questionnaire is completed by the child’s parents. Anthropometric measurements occur at V2, V3, and V4. Compliance is monitored in a diary, specifically developed for the study purpose. Compliance data are checked at V3 and V4. Adverse reactions are recorded as well during V3 and V4.

### Data collection

All data will be recorded by trained clinical investigators and/or by the study site coordinator using an electronic case report form (eCRF Ennov EDC^®^, Ennov 33270 Floirac, France; https://ecrf.chru-lille.fr/EnnovClinical).

Data safety and security measures will be taken into account for the different study sites (restricted staff access, password protection, firewall, and virus spyware protection). To ensure the data quality, a study monitor from the trial sponsor will verify and cross-check all data against the investigator’s source document records. The essential data necessary for monitoring the primary and secondary endpoints has been identified and will be managed at regular intervals throughout the trial by the data management team of the Data Management Department of Lille University Hospital by using the predefined rules. In case of discrepancies, queries will be sent to the investigator and study site coordinator for resolution.

### Statistical analysis

The variation of the Assisting Hand Assessment (AHA) from baseline to 6 months is the primary outcome. Secondary outcomes are the Quality of Upper Extremity Skill Test (QUEST), the Pediatric Quality of Life Inventory 3.0 Cerebral Palsy module (PedsQL™ 3.0 CP Module, Parents Report), SEP parameters (N13-N20 interval, P14-N20 interval, ratios of delay conduction, amplitudes of N20-P27 and amplitude ratio), neuro-orthopedic examination (results of the second examination, 6 months later, scored in comparison with the first examination on a 0 to 3 scale: 0 = normal range of motion, 1 = lower anomaly, 2 = stable anomaly, 3 higher anomaly).

Statistical analyses will be independently performed by the Biostatistics Department of Lille University. Data will be analyzed using the SAS software (SAS Institute Inc, Cary, NC, USA), and all statistical tests will be performed with a 2-tailed alpha risk of 0.05. Baseline characteristics will be described for each group, categorical variables will be expressed as frequencies and percentages, and quantitative variables will be expressed as means and standard deviation in case of normal distribution or medians (interquartile range) otherwise normality of distributions will be assessed graphically and by using the Shapiro-Wilk test. No formal statistical comparisons of baseline characteristics will be done; clinical importance of any imbalance will be noted. For the primary analysis, both analyses (ITT and per-protocol) will be considered to support the conclusion of non-inferiority [45]. ITT population includes all randomized participants based on their original group of randomization. Per-protocol population includes all randomized patients excluding those with major protocol violations: patients who do not complete the rehabilitation procedure allocated after randomization, discontinuation for lack of compliance or personal reasons, adverse reaction. For the secondary objectives, we will use only the ITT population.

#### Primary outcome

The variation of the Assisting Hand Assessment (AHA) from baseline to 6 months will be compared between the two treatment groups using the constrained longitudinal data analysis (cLDA) model proposed by Liang and Zeger [46] including the same fixed and random effects as in the primary efficacy model. This model will be used in view of the potential advantages of the cLDA compared to the conventional longitudinal analysis of covariance (ANCOVA) model [47]. In the cLDA, both the baseline and post-baseline values will be modeled as dependent variables using a linear mixed model (using an unstructured covariance pattern model), and the true baseline means will be constrained to be the same for the 2 treatment groups. The between-group mean differences in 6-months change in AHA will be estimated by the time-by-arm interaction as treatment effect size. In absence of missing data, the cLDA model provides the same point estimates of treatment differences than the ANCOVA model. Nevertheless, cLDA presents two main advantages with regard to the ANCOVA model: first, the estimated variance from the ANCOVA model is always greater than or equal to that from the cLDA model and consequently cLDA model provides more appropriate confidence interval of estimates. Second, if there are missing values, the cLDA model allows to provide unbiased estimates of the treatment effect under the MAR (missing at random) assumption, which is not the case for the ANCOVA model. If the assumption of normality of model residuals is not satisfied (even after log-transformation), non-parametric analysis will be used; absolute changes between baseline and 24 h will be calculated and compared between the 2 treatments groups using non-parametric analysis of covariance adjusted for baseline values [48].

Missing data (whatever the reason) will be handling using the multiple imputation procedure [49,50]. Imputation procedure will be performed using main baseline characteristics and treatment group under missing at the random assumption by using regression switching approach (chained equation with *m* = 20 imputations) with a predictive mean matching method for continuous variables, logistic regression models (binary, ordinal or polynomial) for categorical variables. Imputation procedure will be performed using the baseline characteristics and allocated group. Treatment effect estimates obtained in multiple imputed data sets will be combined using the Rubin’s rules [51]. Complete case analysis will be performed as a sensitivity analysis [52].

#### Secondary outcomes

The variation from baseline to 6 months of the Quality of Upper Extremity Skill Test (QUEST), the Pediatric Quality of Life Inventory 3.0 Cerebral Palsy module (PedsQLTM 3.0 CP Module, Parents Report) and the SEP parameters will be analyzed using the cLDA model. For the neuro-orthopedic examination, we will use a chi-square test or Fisher exact test if appropriate. Details the type of trouble will be described in each group without formal statistical comparison.

## Discussion

The PROPENSIX study is the largest sample size RCT with double-blinding and placebo device, aiming to assess the efficacy of a PGT using compressive dynamic Lycra^®^ sleeve on bimanual performance in children with UCP aged 5 to 10.

To our knowledge, it is the first RCT aiming to involve 100 participants for the evaluation of PGT using Lycra^®^ sleeves in cerebral palsy. It is also the first large sample RCT to be conducted with participants and investigators being blinded regarding the intervention, thanks to a placebo device. A recent systematic review conducted by Almeida et al. [53] analyzed 13 studies covering 4 different types of Lycra^®^ garments; 6 studies were RCT with small sample size. The low statistical power of previous studies is limiting conclusions for clinical practice*.* Martins et al. [54] included 4 RCT in a meta-analysis, totalizing 110 subjects. It demonstrates a significant but small effect size on Growth Motor Function Measure (GMFM) at post-treatment, with moderate heterogeneity between trials. Moreover, the PROPENSIX protocol follows recommendations from Martins et al. [54] to use valid and reliable measures that assess all domains of ICF.

Another strength of the present study relates to the splint compliance and tolerance. We believe that the sleeve type of Lycra^®^ garment would bring a good acceptability and tolerance because it does not cover a large part of the body and lets the fingers free. Adverse effects are expected to be minor. Parent’s complaints concern the discomfort associated with more covering devices. In this protocol, we propose to continue wearing for at least 3 h a day, for a total of 6 months. The usual rehabilitation program of the child does not undergo major modifications. The child does his/her usual activities, rhythmed by the usual environment, in a context of confidence.

We wanted the major outcome to represent what we really want to enhance when we manage the rehabilitation of children with UCP. Bimanual performance, which designates what the child really does in an ecological context, is the principal measure outcome, assessed by the AHA.

The results of the SEP as a secondary outcome should emphasize the link between somatosensory dysfunction and motor ability. It could bring interesting arguments to discuss whether enhancement of motor function is subtended by enhancement of somatosensory function.

The age of the study population can be discussed. The 5–10-year-old frame allows a better involvement of the child in the rehabilitation program. However, fine prehension acquisition occurs earlier and it could be interesting to conduct similar studies in younger children. Regarding other potential limitations of the present protocol, we can cite the absence of long-term evaluation and the lack of a goal attainment specific scale, such as Goal Attainment Scaling (GAS) or Canadian Occupational Performance Measure (COPM).

## Conclusion

PROPENSIX study will provide multidimensional arguments about the efficacy of a Pressure Garment Therapy using compressive dynamic Lycra^®^ sleeve in children with UCP. This RCT is the first large sample size randomized controlled trial aiming to evaluate this type of therapy in this population. Improvement of children’ bimanual performances at the end of the 6-month wear period could strengthen evidence regarding this therapy. The results of the secondary outcomes could bring interesting arguments to discuss Lycra^®^ sleeve action on mobility, tonus, and sensory impairments in children with UCP.

## Data Availability

The final trial dataset generated during the study is the property of the study sponsor (Lille University Hospital). The final trial dataset will be firstly available to biostatisticians involved in the study. The final trial dataset will be secondly available to all involved researchers if needed and based on the review of the request. A disclosure of contractual agreements has been signed by all involved researchers under the responsibility of the study sponsor (Lille University Hospital).
